# Preparation and Properties of Mo Coating on H13 Steel by Electro Spark Deposition Process

**DOI:** 10.3390/ma14133700

**Published:** 2021-07-01

**Authors:** Wenquan Wang, Ming Du, Xinge Zhang, Chengqun Luan, Yingtao Tian

**Affiliations:** 1 Key Laboratory of Automobile Materials, School of Materials Science and Engineering, Jilin University, Changchun 130025, China; wwq@jlu.edu.cn (W.W.); mingdu19@mails.jlu.edu.cn (M.D.); chengqun16@mails.jlu.edu.cn (C.L.); 2 Department of Engineering, Lancaster University, Bailring, Lancaster LA1 4YW, UK; yingtao.tian@manchester.ac.uk

**Keywords:** electro spark deposition (ESD), Mo coating, H13 steel, microhardness, wear resistance, corrosion resistance

## Abstract

H13 steel is often damaged by wear, erosion, and thermal fatigue. It is one of the essential methods to improve the service life of H13 steel by preparing a coating on it. Due to the advantages of high melting point, good wear, and corrosion resistance of Mo, Mo coating was fabricated on H13 steel by electro spark deposition (ESD) process in this study. The influences of the depositing parameters (deposition power, discharge frequency, and specific deposition time) on the roughness of the coating, thickness, and properties were investigated in detail. The optimized depositing parameters were obtained by comparing roughness, thickness, and crack performance of the coating. The results show that the cross-section of the coating mainly consisted of strengthening zone and transition zone. Metallurgical bonding was formed between the coating and substrate. The Mo coating mainly consisted of Fe_9.7_Mo_0.3_, Fe-Cr, FeMo, and Fe_2_Mo cemented carbide phases, and an amorphous phase. The Mo coating had better microhardness, wear, and corrosion resistance than substrate, which could significantly improve the service life of the H13 steel.

## 1. Introduction

H13 mold steel is mainly used for casting, extrusion, hot forming, and plastic molding applications due to its excellent hot-cold manufacturing properties, dimensional stability, and impact toughness resistance [1,2,3,4]. H13 mold steel is usually manufactured by casting and forging processes [5], which result in coarse carbides formation because of its low cooling and solidification rate [6,7]. The surface performance will be significantly reduced if H13 mold steel is exposed to high temperature for a long time [8,9,10]. Nowadays, some researchers have proposed to refine carbides by forging and rolling [11]. Although some achievements have been made, the problem has not been fundamentally solved. Moreover, mold steel can be easily damaged or fail due to wear and electrochemical corrosion, which vastly shortens its service life. To further improve the mold’s service life, it is necessary to strengthen mold’s wear resistance and corrosion resistance [12,13]. The appropriate strengthening methods of improving wear and corrosion performance have become a hotspot on the surface modification of hot work molds [14]. Therefore, many scholars from various countries have conducted extensive research on mold’s surface modification. Kong et al. [15] prepared WC-12Co coatings to improve H13 mold steel’s wear resistance and electrochemical corrosion performance. Żórawski et al. [16] revealed that the nanostructured WC-12Co composite coating has a denser structure and higher abrasive resistance than conventionally sprayed coatings. Meng et al. [17] investigated the mechanical properties and microstructures of H13 steel processed by a bionic laser surface alloying with different amount of TiC addition. With the increase of TiC fraction, the laser alloying zone’s microstructure was refined and the microhardness was increased. Salmaliyan et al. [18] studied the effect of ESD process parameters on WC-Co coating with substrate of H13 steel. The results indicate that the crack propagation properties of the coating at low spark energy are different from those at high spark energy. Compared with other coating preparation technologies, ESD has attracted more interest as a promising surface technique for engineering materials [19,20,21]. ESD technology has unique advantages in preparing coatings such as WC-Co, FeNiCrBSiC-MeB2, Ti-Al intermetallic, CoCrFeNiMo High-Entropy Alloy, Ti6Al4V, and Cr coating due to its simple equipment, flexible operation, low cost, ecological greenness, wide adaptation range, and low heat input [22,23,24,25,26,27,28].

Mo is important to the industry due to its high-melting-point, high thermal conductivity, and low thermal expansion coefficient [29]. Moreover, Mo is an excellent coating metal in surface modification due to its superior metallurgical bonding with various metals and alloys. Existing researchers mostly use thermal spraying technology to prepare Mo coating. The only problem of thermal spraying technology is to have a high degree of oxidation [30]. In the present study, Mo coating was fabricated on the H13 steel using ESD technology to obtain better properties (microhardness, wear resistance, and corrosion resistance) than substrate. Properties of Mo coating with different ESD process parameters were investigated in detail.

## 2. Materials and Experimental Methods

### 2.1. Materials

In this study, the H13 steel was chosen as the substrate with sizes of 10 mm × 10 mm × 5 mm. The chemical compositions of H13 steel are shown in Table 1. The specimens were washed with acetone to remove oil and then ground with 600# sandpaper to remove oxides.

### 2.2. Coating Preparation

**Figure 1:**
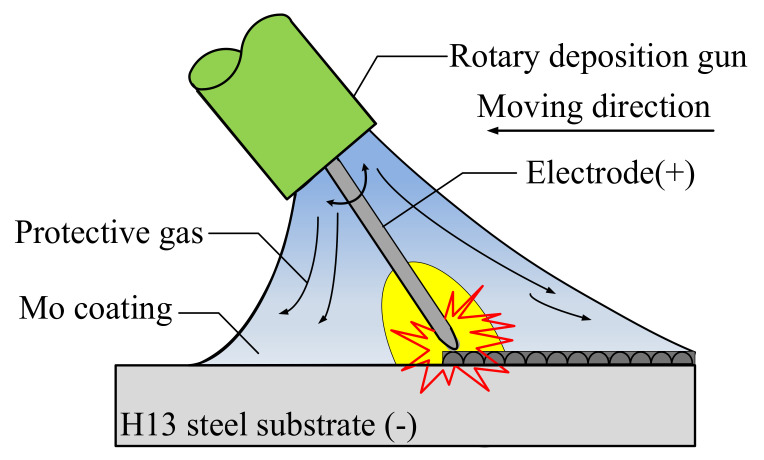
Schematic diagram of preparing Mo coating on H13 steel by ESD.

The DZS-1400 ESD system (Institute of Surface Engineering Technology, Chinese Academy of Agricultural Mechanization Sciences, Beijing, China) was used to prepare the Mo coating on the substrate. Figure 1 shows the schematic diagram of preparing Mo coating on H13 steel by ESD process. Mo was used as the electrode. The electrode was cut into a cylinder with diameter of 3 mm. The inclination angle between the electrode and the substrate was 45 degrees. The rotation speed and moving speed of the processing gun were 1500 r/min during ESD. The shielding gas was argon with a flow rate of 8 L/min. The main processing parameters for ESD are presented in Table 2.

### 2.3. Microstructure and Morphology Testing

The microstructure and wear scar morphology and the chemical compositions were observed and analyzed by scanning electron microscope (SEM, TESCAN VEGA3, Czech Republic) equipped with an energy dispersive spectrometer (EDS). The roughness and three-dimensional morphology of the coating were tested by the OLS3000 (China) laser confocal microscope. Roughness was evaluated quantitatively by arithmetical mean deviation of profile Ra. The phase constitutions were determined via the X-ray diffractometer (XRD) machine (D/Max2500Pc, Japan) using Cu Kα radiation.

### 2.4. Microhardnes and Wear Resistance Testing

**Figure 2:**
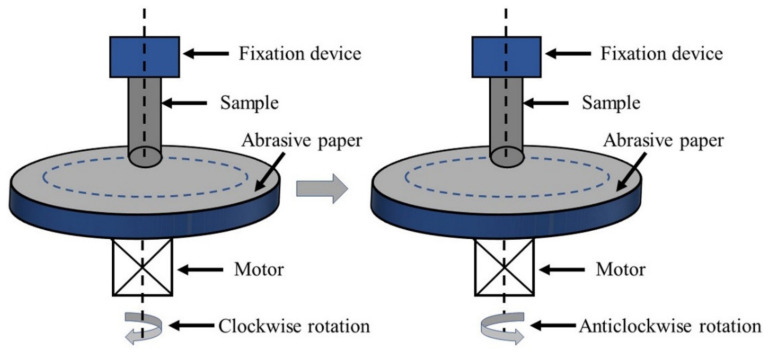
Schematic of friction and wear.

The microhardness of the coating was tested by the HVS-1000 microhardness machine (Beijing Times Guangnan Testing Technology Co., Ltd, China) with a load of 100 g and dwell time of 10 s. The wear resistance of the coating was investigated for the low-speed rotation by the ML-100 abrasive wear test machine (Jinan Jingcheng Test Technology Co., Ltd, Jinan, China). Figure 2 shows the schematic of friction and wear. The silicon carbide sandpaper of 2000# was used as friction material. The travel period of a clockwise rotation and an anticlockwise of the testing machine is taken as the wear time. The load of abrasive wear test machine is 2~10 N. The mass of samples before and after wear is measured by electronic balance (precision 0.0001). The samples with the same parameters were subjected to three wear tests. The wear weight loss was averaged and recorded.

### 2.5. Electrochemical Corrosion Testing

The electrochemical corrosion performance and corrosion mechanism were studied by the VersaSTAAT3 electrochemical corrosion workstation (Guangzhou Beituo Science and Technology Co., Ltd, Guangzhou, China) with a three-electrode system. The saturated calomel electrode was used as the reference electrode and the platinum black electrode as the auxiliary electrode. The potential scanning range was −1.5 V~1 V with the scanning speed of 45 mV/min, and the corrosion medium was 3.5 wt.% NaCl solution. Because the sample was a metal material with electrical conductivity, the non-corrosive area had to be coated with a layer of insulating material to prevent other areas from conducting and affecting the test results.

## 3. Results and Discussion

### 3.1. Coating Appearance

**Figure 3:**
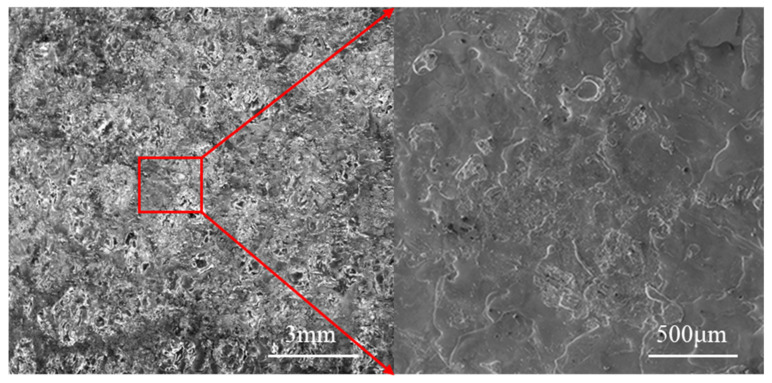
Typical Mo coating appearance.

The Mo coating appearance is shown in Figure 3. The arrowed area is an enlargement view of the area in the red frame. Figure 3 displays the orange peel-like morphology of Mo coating, which is a typical morphology of ESD coating. The coating is formed by fusion and superposition of numerous deposition points during ESD process. As a result, the Mo coating has a rough surface due to the uneven coverage of deposition points.

**Figure 4:**
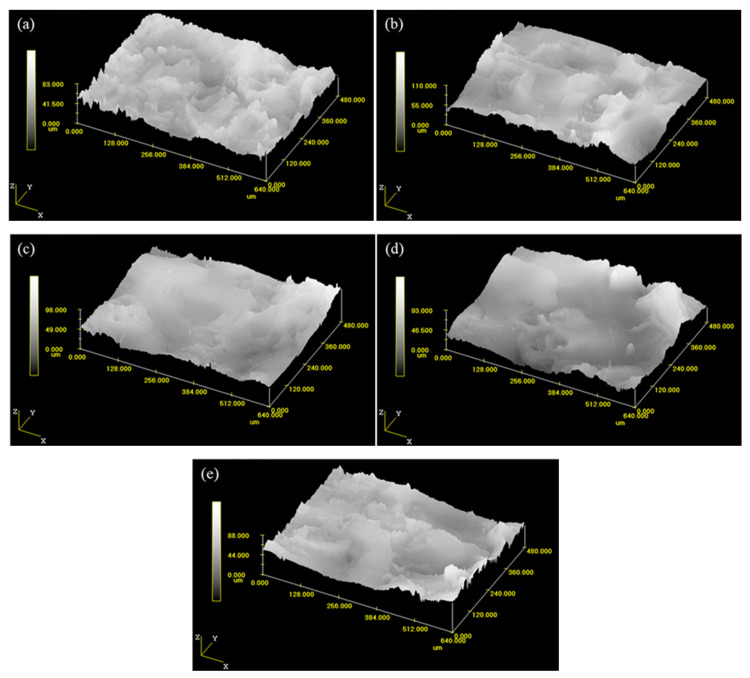
The three-dimensional morphology of coating deposited with different deposition power: (**a**) 100 W (Ra = 2.6 μm), (**b**) 500 W (Ra = 2.9 μm), (**c**) 800 W (Ra = 4.75 μm), (**d**) 1000 W (Ra = 4.35 μm), and (**e**) 1400 W (Ra = 3.8 μm).

**Figure 5:**
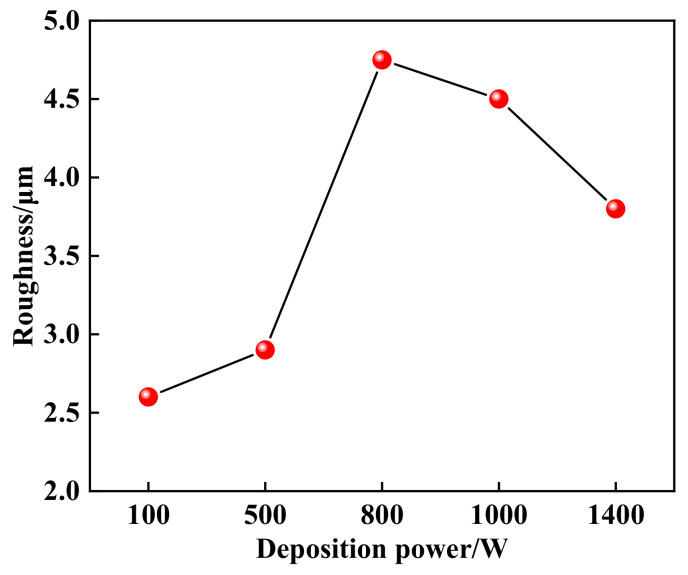
Effect of deposition power on the roughness of coating.

Figure 4 shows the three-dimensional morphology of the coating with different deposition powers. Figure 5 indicates the effect of the deposition power on the roughness of the coating. It can be seen that the roughness of the coating is different. When the deposition power is less than 800 W, the roughness of the coating increases with the deposition power. However, when the deposition power exceeds 800 W, the roughness decreases. The roughness is mainly related to the discharge energy per unit time. During the ESD process, the pulse discharge energy per unit time can be expressed by the following formula:

(1) $$E=P\times f\times t_{on}$$

where *E* is the pulse electric spark discharge energy per unit time, *P* is the deposition power; *f* is the pulse frequency, and *t_on_* is the single pulse discharge time. According to Equation (1), the greater the deposition power *P* is, the greater the pulse spark discharge energy *E* per unit time is. When the deposition power is less than 800 W, the melting amount of the electrode increases with the deposition power. It is difficult to uniformly connect and stack of the deposition points during the deposition process due to the rapid cooling rate of the liquid metal, resulting in the increase of roughness. The melting amount of the electrode increases significantly when the deposition power exceeds 800 W. The flow of liquid metal could fill the concave of the deposited layer, so the roughness of the coating can be reduced.

**Figure 6:**
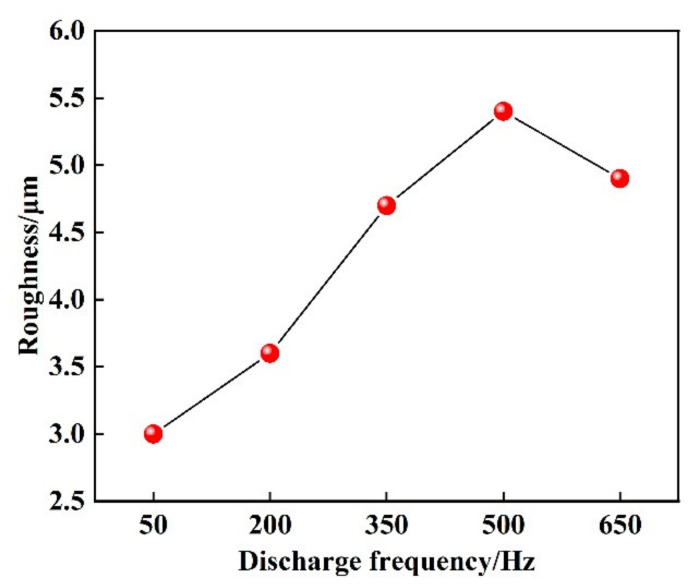
Effect of discharge frequency on the roughness of coating.

Figure 6 shows the effect of the discharge frequency on the roughness of the coating. The results indicate that the roughness of the coating increases with the increase of discharge frequency at first and then decreases slowly. However, the discharge energy used to melt the Mo electrode increases with the increase of discharge frequency. The maximum roughness of the coating is obtained when the discharge frequency is 500 Hz. When the discharge frequency increases from 50 Hz to 500 Hz, the more quickly the Mo deposition points superimpose and the larger of surface height difference is, which results in an increase of roughness. When the discharge frequency increases from 500 Hz to 650 Hz, the amount of liquid metal increases significantly enough to make the liquid metal flow to fill the concave part of the deposition layer. Therefore, the roughness of the coating is reduced.

**Figure 7:**
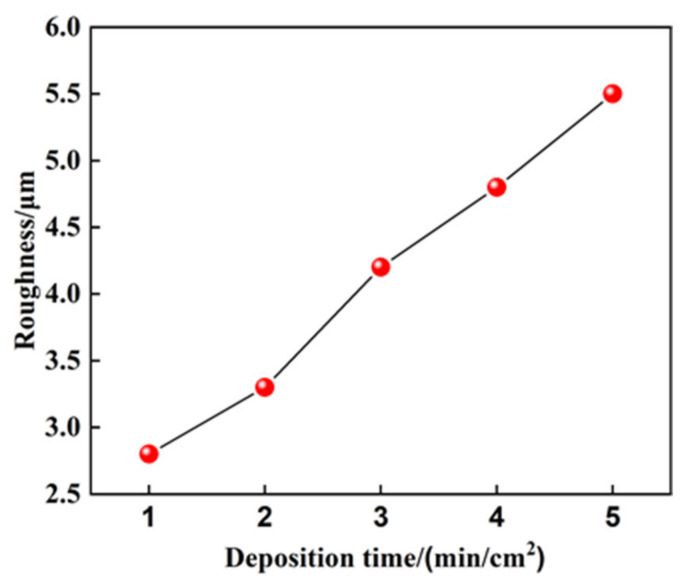
Effect of specific deposition time on the roughness of coating.

The substrate of the 10 mm ×10 mm area is carried out by ESD to prepared Mo coating with different specific deposition time. The specific deposition time is 1~5 min/cm^2^. Figure 7 shows the effect of the specific deposition time on the roughness of the coating. It can be seen from the figure that the roughness of the coating increases with the increase of specific deposition time. The longer the specific deposition time is, the more difficult it is to stack and connect the deposition points evenly and the larger the roughness is. As the specific deposition time increases, the heat input of the coating increases. The thermal stress increases due to multiple rapid melting and rapid cooling. The surface defects of the coating such as shedding, cracks and pores, and the roughness of the deposition layer increase sharply when the specific deposition time exceeds 4 min/cm^2^.

### 3.2. Coating Thickness

**Figure 8:**
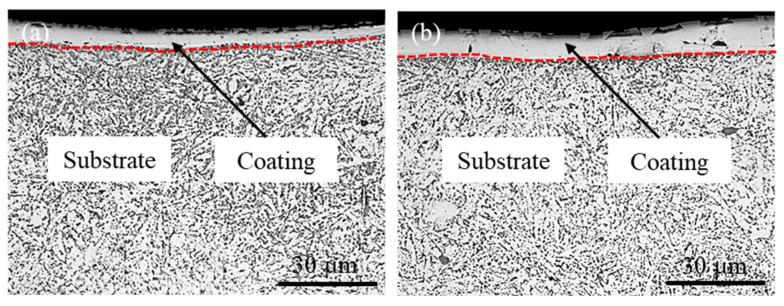

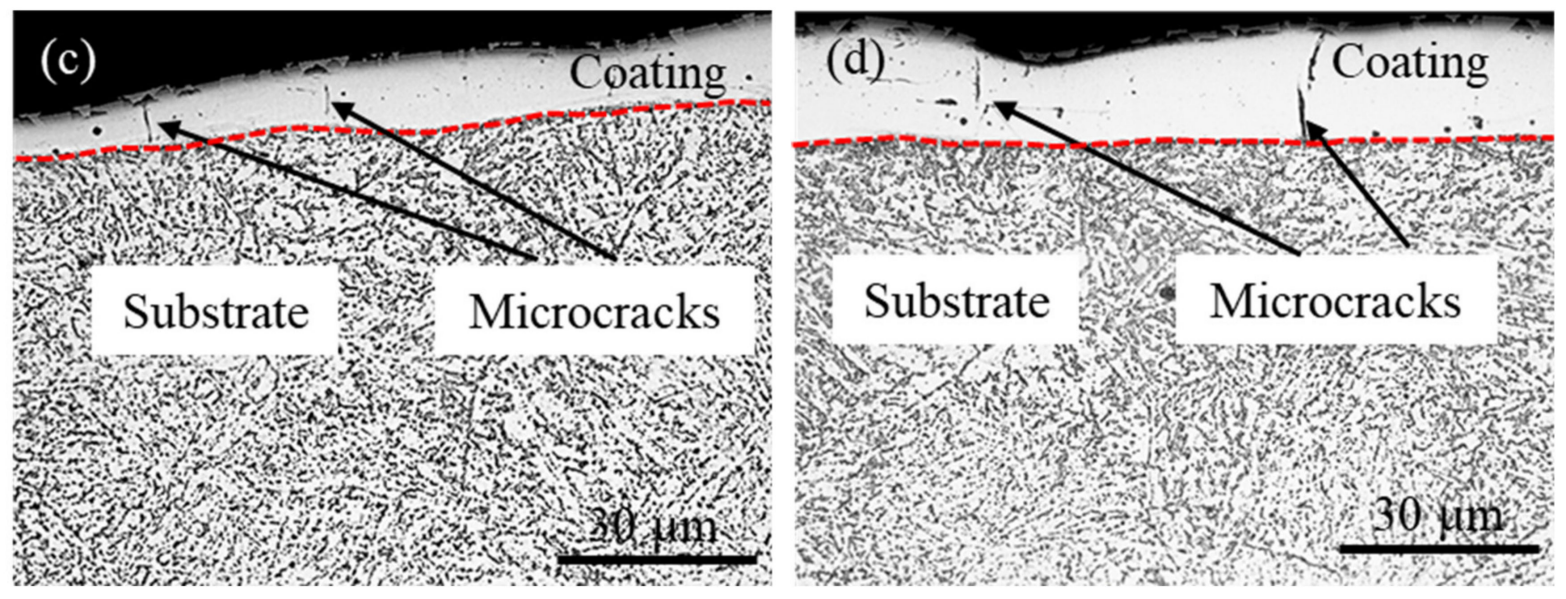
Cross-sectional images of coating: (**a**) 2 min/cm^2^, (**b**) 3 min/cm^2^, (**c**) 4 min/cm^2^, and (**d**) 5 min/cm^2^.

The thickness seriously affects the properties of the coating. The specific deposition time is the crucial parameter that affects the thickness of the coating during ESD. The cross-section images of the coating obtained with different specific deposition time are shown in Figure 8. The thickness of the coating is relatively uniform when the specific deposition time is less than 3 min/cm^2^. The thickness of the coating is uneven when the specific deposition time exceeds 3 min/cm^2^. At the same time, there are microcracks that appear in the coating. There are many reasons for the occurrence of microcracks. On the one hand, the ESD process is a process of instantaneous heating and rapid cooling. If the liquid electrode material that has transitioned to the substrate surface has not completely solidified, the next pulse discharge will arrive. Thereby, thermal stress is generated in the material. On the other hand, the ESD process is accompanied by thermal diffusion. The heat from the coating surface was diffused along the depth direction of the substrate. Appearance of microcracks in the coating is due to the large temperature gradient during the ESD process.

**Figure 9:**
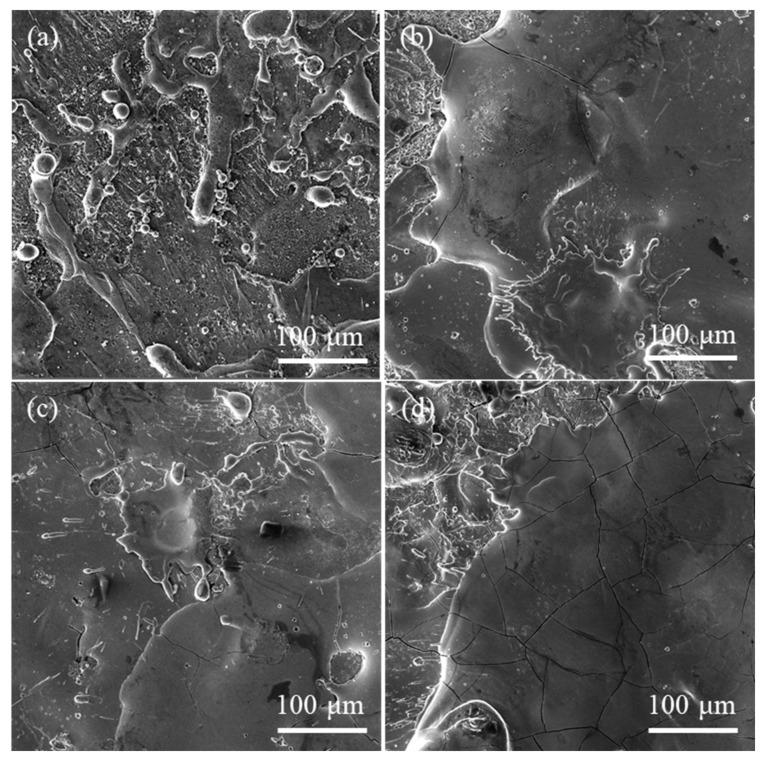
Frontal images of coating: (**a**) 2 min/cm^2^, (**b**) 3 min/cm^2^, (**c**) 4 min/cm^2^, and (**d**) 5 min/cm^2^.

The frontal images of coating fabricated by different processing parameters are shown in Figure 9. The coatings display a splash pattern. With the increasing of the specific deposition time, the coating surface has more microcracks.

**Figure 10:**
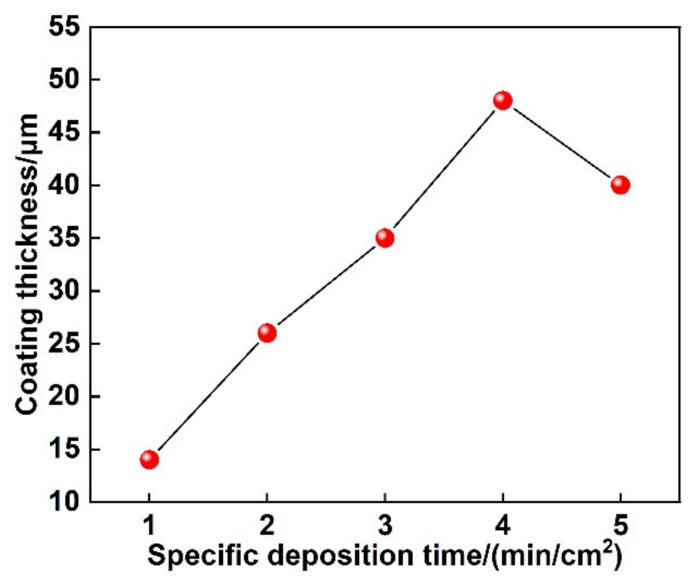
Effect of specific deposition time on the thickness of coating.

Figure 10 shows the effect of specific deposition time on the thickness of the coating. The thickness of the coating increases with specific deposition time when the specific deposition time is less than 4 min/cm^2^. However, the thickness of the coating decreases when the specific deposition time increases to 5 min/cm^2^. Due to the long specific deposition time, the coating undergoes many times rapid melting and cooling processes. Therefore, the surface of the coating produces thermal stress. It is shown in Figure 8c,d that the microcracks defects are formed in the coating. These defects cause the coating to fall off, and then the thickness of the coating decreases significantly. The plasticity and toughness of the coating can be decreased.

**Figure 11:**
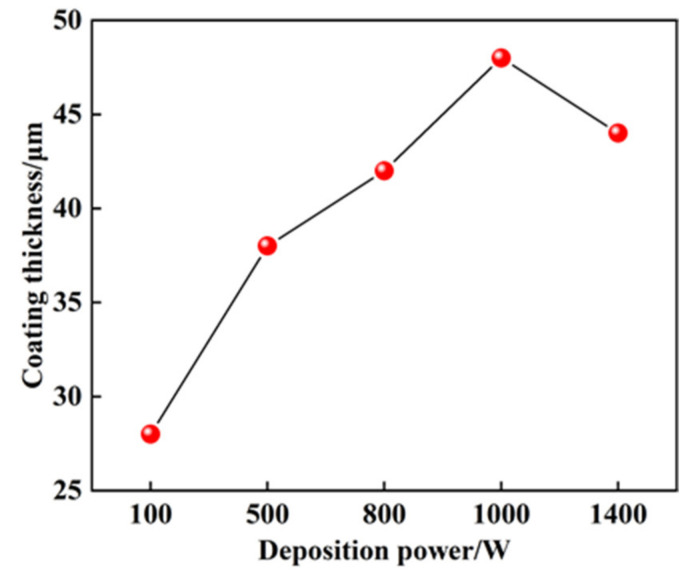
Effect of deposition power on the thickness of coating.

The effect of deposition power on the thickness of the coating is shown in Figure 11. The thickness of the coating first increases and then decreases with the increase of the deposition power. The maximum thickness of the coating is obtained when the deposition powers is 1000 W. The energy of electro spark discharge and melting rate of the electrode increase with the increase of the deposition power when the deposition powers are 100~1000 W. Therefore, the coating thickness increases gradually with the increase of deposition power. However, the melting rate of the electrode increases considerably when the deposition power exceeds 1000 W, resulting in the increase of cooling time of molten Mo, and the splatters of molten Mo increase with the rotation of the electrode. In addition, due to the increase of high thermal stress, it is easy for the coating to fall off and crack, so the coating thickness decreases with the improvement deposition power.

**Figure 12:**
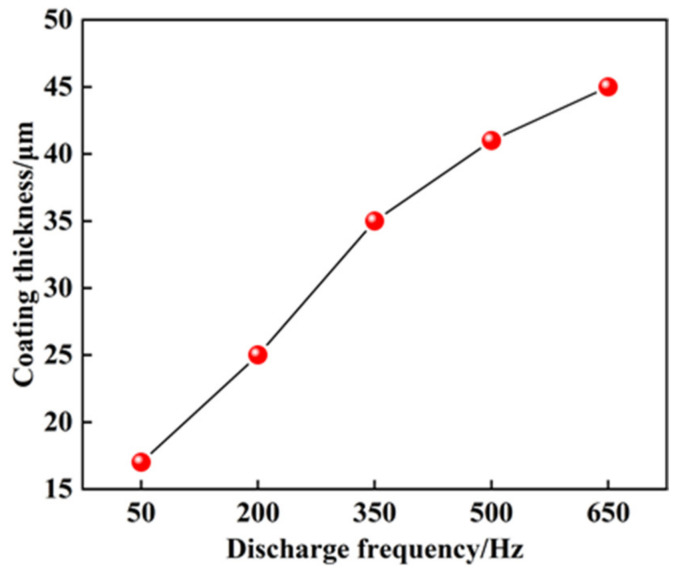
Effect of discharge frequency on the thickness of coating.

Figure 12 shows the relationship between the discharge frequency and the coating thickness. The discharge frequency was set to 50 Hz, 200 Hz, 350 Hz, 500 Hz, and 650 Hz as variable parameters to prepare the coating. The results show that the thickness of the coating increases with increasing discharge frequency. According to Equation (1), the discharge energy used to melt the electrode increased with the increase of discharge frequency, and then the thickness of the coating increased with the same specific deposition time. The electrode melting rate increases with the increase of discharge frequency when the discharge frequency exceeds 350 Hz. However, the thickness of the coating growth rate also slows down due to the slow growth of melting rate and melting amount of the electrode.

### 3.3. XRD and Chemical Compositions Analysis of the Typical Coating

The thickness and roughness of the coating are important indexes to evaluate the quality of the coating. The effects of the deposition power, discharge frequency, and specific deposition time on the coating quality were studied, and the optimal parameters were obtained as follows: the deposition power, discharge frequency, and specific deposition time were 1000 W, 350 Hz, and 3 min/cm^2^, respectively. Finally, the Mo coating with 35 μm thickness and about 5.0 μm roughness was prepared without severe cracks.

**Figure 13:**
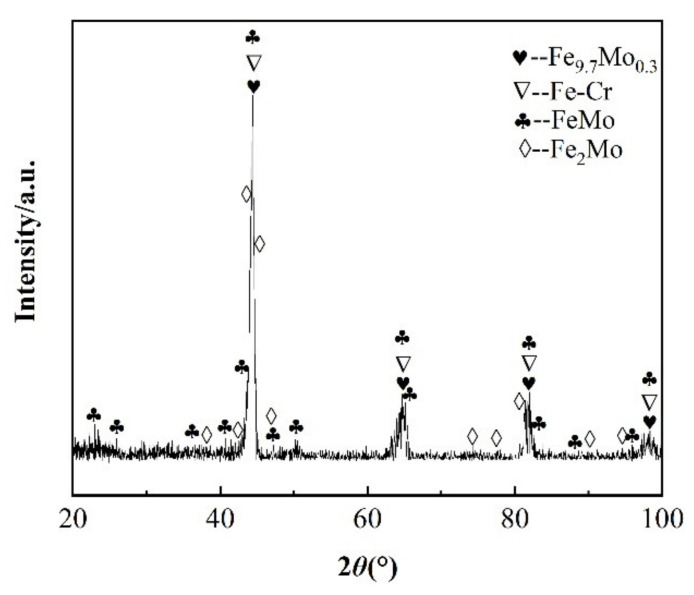
XRD pattern of coating fabricated under 1000 W/350 Hz/(3 min/cm^2^).

The XRD pattern of the coating fabricated under 1000 W/350 Hz/(3 min/cm^2^) by the ESD process is shown in Figure 13. The results show that the coating was composed of Fe_9.7_Mo_0.3_, Fe-Cr, FeMo, and Fe_2_Mo cemented carbide phases. Jia Delong et al. obtained similar XRD analysis results at the plasmas sprayed Mo coating on stainless steel substrate [31]. The Fe_9.7_Mo_0.3_, FeMo, and Fe_2_Mo phases were formed by the metallurgical reaction between the Mo electrode material and the H13 steel substrate during the ESD process. It is beneficial for good mechanical properties that the coating was formed by the metallurgical bond between the Mo coating and the H13 steel substrate [32]. In addition, the diffraction peaks of the Mo coating were scattered and miscellaneous, indicating that the coating contained a large number of amorphous phase structure.

**Figure 14:**
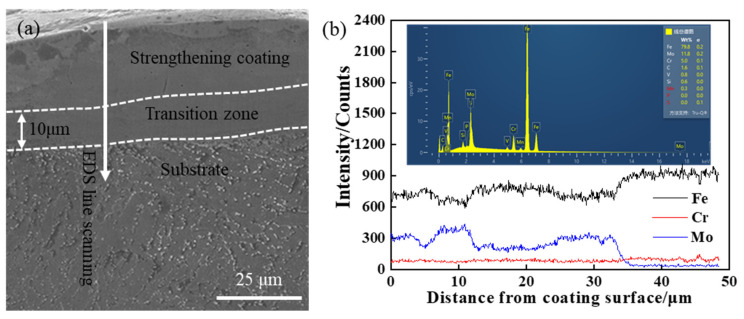
Morphology cross-section and elements distribution of coating fabricated under 1000 W/350 Hz/(3 min/cm^2^): (**a**) the cross-section of coating and (**b**) line scanning results of elements distribution.

**Figure 15:**
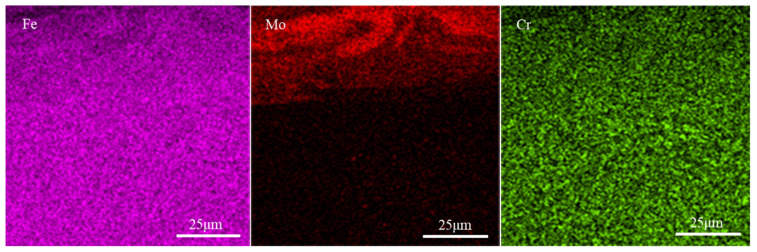
Map scanning results of elemental distribution of coating fabricated under 1000 W/350 Hz/(3 min/cm^2^).

Figure 14 shows the cross-section image and line scanning results of elements distribution of the coating. The elemental mapping of Figure 14a is shown in Figure 15. The cross-section of the coating mainly consists of the strengthening zone and transition zone. The thickness of the coating is about 35 μm, and that of the transition zone is about 10 μm. There are continuous and close bonds between the coating and the substrate, and there are no cracks and pores at the interface. The line and map scanning results of elements distribution indicate that the coating is mainly composed of Mo, Fe, and Cr elements. The contents of Fe and Cr elements gradually increase from the coating top surface to the substrate, while the content of Mo element gradually decreases. As shown in Figure 15, the enrichment of Mo element in the upper part of the coating is pronounced, while there are also a few Mo element in the substrate. The results also indicate that coating and substrate elements diffuse each other, and the metallurgical bond is formed between the coating and substrate. During the ESD process, there would be a small amount of Fe and Cr elements in the coating due to a small amount of substrate melted by electric erosion. Besides, the coating temperature is higher than the substrate, which is beneficial to diffusing the Fe and Cr elements into the coating. Simultaneously, this is also the reason for the formation of the transition zone.

### 3.4. Properties Analysis of the Typical Coating

#### 3.4.1. Microhardness

**Figure 16:**
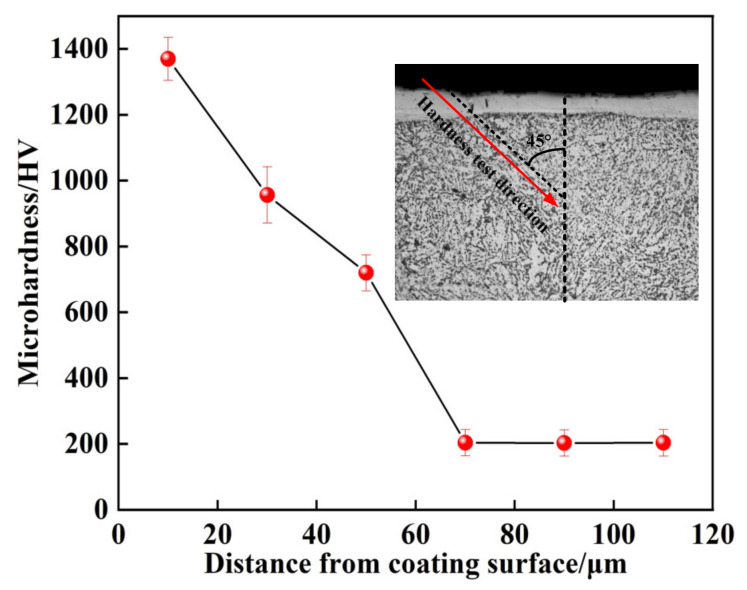
Hardness distribution of coating fabricated under 1000 W/350 Hz/(3 min/cm^2^).

Microhardness tests are conducted on the coating cross-section, and Figure 16 shows the hardness distribution of the coating. The results indicate that the microhardness of the coating gradually decreased from the coating top surface to the substrate. The highest microhardness of the coating value is 1369.5 HV, which is about 6.7 times the substrate. Due to the thin thickness of the coating, the element content in the micro zone of the coating changes greatly, so the microhardness gradient of the coating changes greatly. The analysis shows that during the ESD process, the Mo electrode has a metallurgical reaction with an H13 steel substrate, and the cemented carbide phase is generated on the surface of the H13 steel substrate. At the same time, the amorphous structure is formed inside the coating. The amorphous phase and the cemented carbide phase improve the microhardness and strengthen the bearing capacity of the coating. However, the microhardness distribution of the coating shows obvious gradient, which is beneficial to improve the friction and wear capacity of the coating.

#### 3.4.2. Wear Resistance

**Figure 17:**
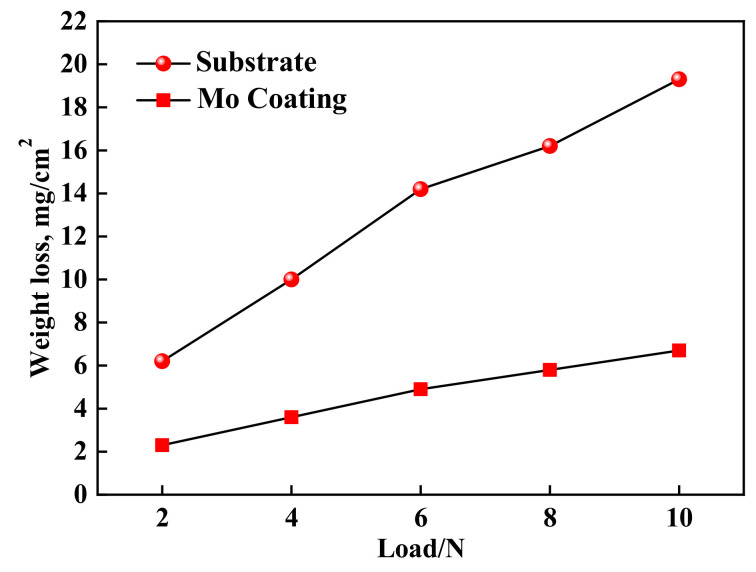
Curves of wear resistance for substrate and coating.

**Figure 18:**
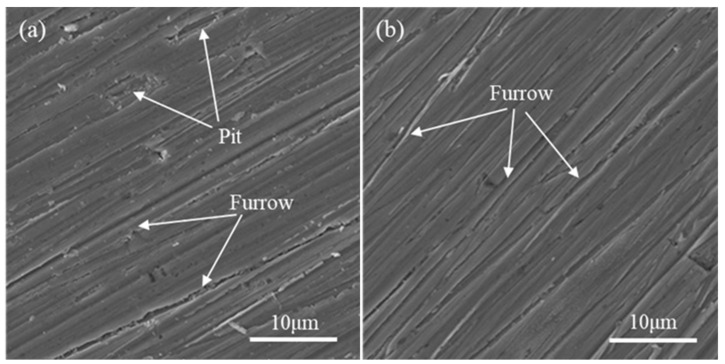
Wear scar morphology: (**a**) substrate and (**b**) coating.

The coating and the substrate samples are conducted at room temperature by the abrasive wear test, and the weight losses of the samples under different loads are measured to evaluate the wear resistance. The wear resistance curves of substrate and Mo coating samples are shown in Figure 17. Under the same load, the weight loss of the coating is less than that of the substrate. With the increase of the load, the weight loss of the coating increases slightly and gently, but the weight loss of substrate increases sharply. According to the weight loss under the same load condition, the wear resistance of the coating is about seven times higher than that of the substrate. The good wear resistance of the coating corresponds to the defect-free and high microhardness of the coating and the good metallurgical bonding between the substrate and coating. In addition, there are a large number of cemented carbides in the coating, which are dispersed in the coating, thus significantly improving the wear resistance of the coating. The wear scar morphology of substrate and coating samples is shown in Figure 18. For the wear sample of the substrate, the furrow-shaped wear scars are deep and wide, and there are many pits formed by the material peeling off locally. However, the furrow-shaped wear scars on the coating surface are smoother and shallower, and there are no pits, which is consistent with the microhardness and weight loss results.

#### 3.4.3. Corrosion Resistance

**Figure 19:**
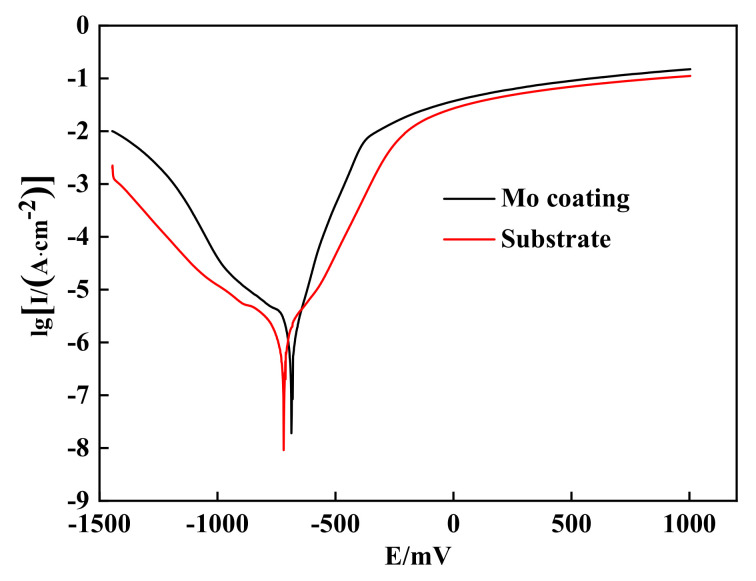
Polarization curves of substrate and coating.

The polarization curves of substrate and coating are obtained by electrochemical etching as shown in Figure 19. It can be seen that the sample of the substrate self-corrosion potential is −682 mV, and the self-corrosion current density is 2.01 × 10^−3^ A/m^2^. The coating’s self-corrosion potential was −621 mV, and the self-corrosion current density is 4.89 × 10^−4^ A/m^2^. Compared with the substrate, the self-corrosion of the coating potential increased by 9%, and the self-corrosion current density was 24% of the substrate, which meant that the corrosion rate of the coating was significantly reduced. The corrosion resistance of the coating was much better than that of the substrate due to Mo’s excellent corrosion resistance.

## 4. Conclusions

In this study, the ESD process was used to prepare Mo coating on the H13 steel substrate, and the properties of the coating were investigated. The main conclusions are as follows.

(1) The Mo coating without defects was successively prepared on the H13 steel substrate by ESD process. The effect of main parameters on the thickness and roughness of the coating was studied. The well main parameters were obtained as follows: the deposition power, discharge frequency, and specific deposition time were 1000 W, 350 Hz, and 3 min/cm^2^, respectively.
(2) The Mo coating was mainly composed of Fe_9.7_Mo_0.3_, Fe-Cr, and FeMo, Fe_2_Mo cemented carbide phases and amorphous phase. The coating cross-section mainly consisted of the strengthening zone and transition zone. The metallurgical bonding was formed between the coating and substrate.
(3) The microhardness of the coating distribution showed a noticeable gradient, and the microhardness gradually decreased from the coating top surface to the substrate. The wear resistance of the coating was about seven times higher than that of the substrate. The amorphous phase and cemented carbide phase improved the microhardness and strengthened the bearing capacity of the coating.
(4) The self-corrosion of the coating potential increased by 9%, and the self-corrosion current density was 24% of the substrate. The corrosion resistance of the coating was much better than that of the substrate due to Mo’s excellent corrosion resistance by ESD technology preparation.

## Figures and Tables

**Table 1:** Chemical compositions of H13 steel. (wt%).

Cr	Mo	Si	V	C	Mn	S	P	Fe
5.0	1.30	0.95	0.92	0.40	0.35	0.05	0.03	Bal.

**Table 2:** ESD process parameters.

Deposition Power	Output Voltage	Discharge Frequency	Specific Deposition Time
100~1400 W	100 V	50~650 Hz	1~5 min/cm^2^

## Data Availability

The data presented in this study are available on request from the corresponding author.
